# Evidence of an Antimicrobial-Immunomodulatory Role of Atlantic Salmon Cathelicidins during Infection with *Yersinia ruckeri*

**DOI:** 10.1371/journal.pone.0023417

**Published:** 2011-08-09

**Authors:** Andrew Bridle, Elizabeth Nosworthy, Mark Polinski, Barbara Nowak

**Affiliations:** National Centre for Marine Conservation and Resource Sustainability, University of Tasmania, Tasmania, Australia; INRA, France

## Abstract

Cathelicidins are a family of antimicrobial peptides that act as effector molecules of the innate immune system with broad-spectrum antimicrobial properties. These evolutionary conserved cationic host-defence peptides are integral components of the immune response of fish, which are generally believed to rely heavily on innate immune defences to invading pathogens. In this study we showed that Atlantic salmon cathelicidin 1 and 2 (asCATH1 and asCATH2) stimulated peripheral blood leukocytes increasing the transcription of the chemokine interleukin-8. Further, functional differences were identified between the two cathelicidins. In the presence of serum, asCATH1 displayed greatly diminished host haemolytic activity, while the constitutively expressed asCATH2 had no haemolytic activity with or without serum. These findings support our hypothesis that fish cathelicidins exert their primary antimicrobial action at the site of pathogen invasion such as epithelial surfaces. Further, we hypothesise that like their mammalian counterparts in the presence of serum they act as mediators of the innate and adaptive immune response via the release of cytokines thus indirectly protecting against a variety of pathogens. We highlight the importance of this immunomodulatory role from the involvement of asCATHs during an infection with the fish pathogen *Yersinia ruckeri*. While we were able to demonstrate *in vitro* that asCATH1 and 2, possessed direct microbicidal activity against the fish pathogen, *Vibrio anguillarum*, and a common gram negative bacterium, *Escherichia coli*, little or no bactericidal activity was found against *Y. ruckeri*. The contribution of either asCATH in the immune response or as a potential virulence factor during yersiniosis is highlighted from the increased expression of asCATH1 and 2 mRNA during an *in vivo* challenge with *Y. ruckeri* . We propose that Atlantic salmon cathelicidins participate in the interplay between the innate and adaptive immune systems via the release of cytokines enabling a more effective response to invading pathogens.

## Introduction

Cathelicidins are a family of cationic peptides that exert antimicrobial activity at physiological concentrations and are thought to be an important component of host immune defence. These gene-encoded ribosomally synthesised antibiotics are produced as prepropeptides containing a highly conserved cathelin-like domain from which they derive their name. The proteolytic removal of the signal peptide and prosequence yields a heterogeneous mature peptide [1]. The mature peptides typically display amphipathic and cationic properties, which aid in bacterial cell membrane disruption [2]. Initially identified in bovine neutrophils during the early 1990s [3] cathelicidins have subsequently been found in a variety of mammals [4]. Once believed to be mammal-specific peptides, cathelicidin genes have since been characterised in the ancient vertebrate, the Atlantic hagfish [5] followed by discovery in rainbow trout [6], chickens [7], Atlantic salmon [2], and other divergent fish species [8], [9], [10] and reptiles [11], [12]. Extensive *in vitro* studies conducted using mammalian cathelicidins have shown that they possess broad antimicrobial activities enabling defence against a range of Gram negative and Gram positive bacteria, fungi, parasites and viruses [13]. More limited studies performed *in vivo* demonstrate the importance of cathelicidins in mammalian defence mechanisms. The lack of the murine cathelicidin (CRAMP) in CRAMP-knockout mice increases susceptibility to group A *Streptococcus* infections [14]. Likewise the human cathelicidin LL-37 has been shown to have a protective function against several invasive bacterial skin infections [15], [16]. Further, overexpression of LL-37 in a cystic fibrosis xenograft model increased antimicrobial activity and bacterial killing [17].

In addition to their predominant antimicrobial role mammalian cathelicidins possess several other biological activities including the ability to chemoattract neutrophils, monocytes and T cells [18]; support antisepsis via LPS binding abilities [19], and promote angiogenesis and wound healing [20] [21]. The strong antibacterial activity, even against multi-drug resistant strains, and immunomodulatory properties of cathelicidins make them a popular candidate for clinical treatments of infection and disease [21]. Although most of these activities have been characterised in mammals it remains to be seen if cathelicidins of non-mammalian vertebrates such as fish possess similar capabilities other than their direct antimicrobial activity and potential haemolytic activity. In fact research on fish cathelicidins has been predominantly limited to identification, genetic characterisation and tissue expression studies. Although several of these studies identified antibacterial and host haemolytic activities of the cathelicidins only a few investigated expression changes after bacterial infection [22]. While the knowledge of fish cathelicidin functions is limited, the evolutionary status of fish and their greater dependence on innate immune mechanisms to prevent microbial infection [23] make them interesting candidates to study the immune functions of cathelicidins.

While usually considered beneficial, the expression of cathelicidins can also contribute to pathophysiology of diseases. Cathelicidin LL-37 expression is increased in patients with psoriasis and rosacea, but decreased in those with atopic dermatitis. In rosacea, overexpression causes inflammation and abnormal blood vessel growth through cell activation [24]. In psoriasis, it is believed that self RNA or DNA forms complexes with LL-37, which drives auto-inflammatory responses associated with the disease [25], [26]. Similarly low levels of LL-37 in saliva are associated with periodontal disease [27] and have been implicated in chronic ulcers [28].

Although humans and mice have a single cathelicidin, the majority of species possess more than one member of the cathelicidin family. Recently, xenobiotic transfer of a pig cathelicidin gene to mouse skin conferred resistance to group A *Streptococcus* infection [29]. Based on this finding the authors proposed that the duplication and divergence of cathelicidins was beneficial. In Atlantic salmon, two cathelicidin genes have been identified – asCATH1 and asCATH2 [2]. However, unlike the rainbow trout cathelicidins rtCATH1 and 2, genetically characterised by Chang et al. [2], and shown to exhibit antimicrobial activity, neither asCATH1 nor 2 were subject to expression or functional studies. Therefore, having previously identified the involvement of a cathelicidin gene from an unpublished cDNA microarray study of Atlantic salmon challenged with the fish pathogen *Y. ruckeri* we decided to further investigate the role that cathelicidins may play in a non-mammalian species such as Atlantic salmon in combating bacterial infection.

We show that asCATH2 in contrast to asCATH1 was constitutively expressed in healthy uninfected Atlantic salmon and that the expression of both was induced during infection with *Y. ruckeri*. Moreover, the potent antimicrobial activity of both cathelicidins against *E. coli* and the fish pathogen *V. anguillarum* was non-existent when tested against *Y. ruckeri*. As the constitutively expressed asCATH2 displayed no host haemolytic activity and the haemolytic activity of asCATH1 was substantially reduced in the presence of host serum we hypothesised that cathelicidins have an immunomodulatory role. We demonstrate that Atlantic salmon cathelicidins stimulate the expression of the chemokine interleukin-8 and to our knowledge this is the first study demonstrating the immunomodulatory role of cathelicidins in fish.

## Results and Discussion

### Differential tissue gene expression of Atlantic salmon cathelicidins

**Figure 1:**
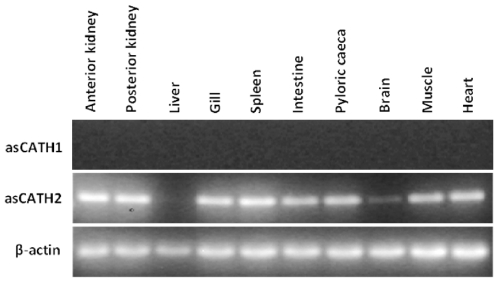
Expression of asCATH1 and 2 in a range of organs from a representative healthy juvenile Atlantic salmon. PCR was performed on cDNA samples from the various organs and 10 µl of each product was visualised on a 1% LB agarose gel. β-actin was included to assess the quality and quantity of cDNA.

Constitutive expression was observed for asCATH2 in the range of organs sampled from healthy Atlantic salmon except the liver. In contrast asCATH1 was not expressed in any of the tissues (Fig. 1). Similar to asCATH1 in the present study rtCATH1 was not detected in any of the tissues analysed until after bacterial infection [2]. Of the salmonid CATH1 clade members identified to date only rtCATH1 and brown trout cathelicidin (btrCATH) tissue expression has been assessed. The btrCATH was constitutively expressed in the anterior and posterior kidney, spleen and stomach, weakly in the brain and skin, but was absent in the muscle and testis [10]. Fish cathelicidins that are closely related to asCATH2 include rtCATH2, Chinook salmon CATH (csCATH), btCATH2, Arctic charr cathelicidin (acCATH) and the grayling cathelicidin (gCATH) [10]. Maier et al. [8] observed constitutive low level expression of acCATH in all tissues tested and Chang et al. [2] found that rtCATH2 was expressed constitutively in all tissues, except the liver of healthy rainbow trout. The patterns observed in fish cathelicidin expression studies show that the constitutive expression of CATH1 cathelicidins is restricted, while CATH2 members are more widely expressed.

### Cathelicidin expression during bacterial challenge from *Yersinia ruckeri*

Yersiniosis is a bacterial disease caused by *Yersinia ruckeri*, which was first isolated in Idaho, USA from rainbow trout during the 1950s [30], [31]. The disease has caused significant losses of farmed salmonids worldwide, predominantly in rainbow trout in the Northern Hemisphere [32]. The Tasmanian serotype O1b of *Y. ruckeri* causes yersiniosis in Atlantic salmon, but not in rainbow trout and brown trout [33]. Having previously identified the involvement of a cathelicidin gene from an unpublished cDNA microarray study of Atlantic salmon challenged with *Y. ruckeri* we further investigated the role of cathelicidins in yersiniosis.

**Figure 2:**
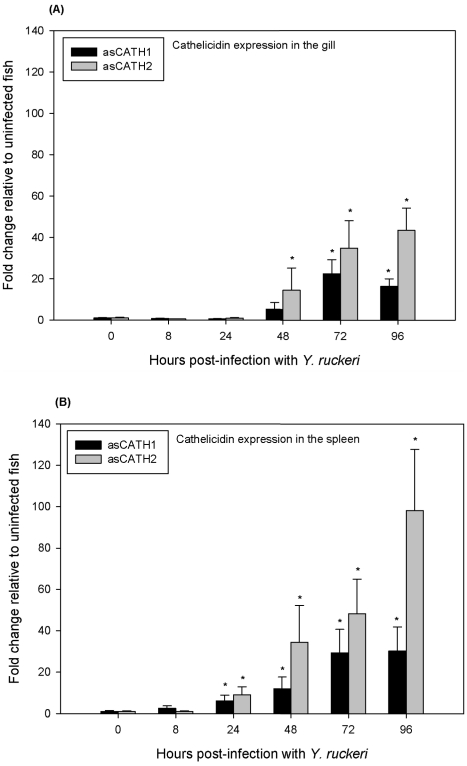
Relative expression of asCATH1 and 2 in the gill (A) and spleen (B) of Atlantic salmon post-infection with *Y. ruckeri*. Gene expression was measured by quantitative real-time PCR. Expression at 8, 24, 48, 72 and 96 h post-infection was compared to expression at 0 h. Each bar represents the mean ± SE of at least 4 fish sampled at each time point. * indicates a significant upregulation of cathelicidin expression compared to expression at 0 h (p<0.05, as assessed by one-way analysis of variance and Dunnett's post-test).

During yersiniosis, the expression of the genes encoding asCATH1 and 2 were upregulated in the gills and spleen of Atlantic salmon. The expression of asCATH2 was upregulated to a greater extent than asCATH1 in both organs at all time points post-infection (Fig. 2). Cathelicidin upregulation has been observed in other fish species after bacterial challenge. Of the salmonids, the rainbow trout and Arctic charr are the only other species for which cathelicidin expression has been analysed after a bacterial challenge. Similar to asCATH1, the expression of rtCATH1 was considerably upregulated in the gill, spleen and head kidney of fish 24 h post-infection with *Aeromonas salmonicida* [6]. A more variable expression of rtCATH2 was found with an earlier upregulation detected in the gill and intestine at 8 h post-infection and a later response at 24 h in the head kidney [2]. Only a small upregulation of rtCATH2 was detected in the spleen of infected fish, which is contrary to the observed expression changes of asCATH2. Overall the relative expression of rtCATH2 was higher than rtCATH1, which supports the observations made for asCATH1 and 2. The rainbow trout beta defensins are another family of AMPs within which different family members exhibit varied expression changes during an infection. For example, the beta defensin 3 (omDB-3) was upregulated 16 fold relative to controls 48 h post-infection with *Y. ruckeri* compared to omDB-1, which increased only 2 fold [34]. Maier et al. [8] investigated cathelicidin expression post-infection with *A. salmonicida* in Atlantic cod and Arctic charr, both of which showed higher expression at 24 h post-infection compared to uninfected fish, which is consistent with observations made in this study [8]. No change in codCATH expression was detected in the spleen, but an increase was seen in the liver, pyloric caeca, intestine and to a lesser extent the gill and skin [8]. The acCATH, which is a member of the salmonid CATH2 clade, gene expression increased in all tissues, particularly the skin, gill, intestine, spleen and kidney [8]. At 5 d post-infection acCATH was highly expressed in all tissues analysed [8], indicating that fish cathelicidin expression may be prolonged during an infection.

Atlantic salmon cathelicidins exhibited extended upregulation during yersiniosis. Both genes encoding asCATH1 and 2 were significantly upregulated at least 96 h post-infection (Fig. 2). The upregulation of asCATH1 did not vary greatly between 96 and 72 h post-infection suggesting expression had peaked; however the asCATH2 gene was further upregulated. For example, in the spleen of infected fish asCATH1 expression was maintained at approximately 30 fold higher than the expression in control fish at 72 and 96 h. Alternatively, asCATH2 expression more than doubled in fold increase from 48 fold at 72 h post-infection to 98 fold at 96 h. In rainbow trout, cathelicidin expression was not analysed beyond 24 h, but rtCATH1 gene expression was highest at 24 h while rtCATH2 expression was variable among the tissues [2]. The expression of rtCATH2 was highest in the gill and intestine at 8 h and had returned to normal levels by 24 h post-infection, which is contrary to observations made for asCATH2. However, investigations in Arctic charr, found that acCATH, another CATH2 group member, was expressed at a high level in all tissues 5 d post-infection, supporting the notion that the CATH2 genes are expressed for a prolonged period post-infection. The codCATHs were also reported to show extended expression remaining significantly upregulated 72 h post-infection with *A. salmonicida*, although expression was much lower than that observed at 24 h [35].

Both asCATH1 and 2 were upregulated to a greater extent and earlier in infection in the spleen compared to the gills (Fig. 2). Other fish AMPs, such as the rainbow trout cathelicidins and β-defensins, the codCATHs and white bass moronecidin, show variable tissue expression of peptides during infection [2], [34], [35], [36]. In an experimental infection of rainbow trout high bacterial loads of *Y. ruckeri* were detected in the gills immediately after infection with a rapid spread to internal organs [37]. At 6 h post-infection the number of bacteria in the spleen peaked, while gill numbers were highest earlier in infection and bacterial counts decreased from 6 to 48 h before increasing again to 72 h, more so in the spleen than gills [37]. Although in rainbow trout, not Atlantic salmon, the higher bacterial load in the spleen during yersiniosis could explain the higher expression of the Atlantic salmon cathelicidins in the spleen compared to the gills.

In addition to prolonged expression, the cathelicidin genes are upregulated well above the expression levels in normal, uninfected fish (Fig. 2). The expression of asCATH2 was upregulated as much as 98 fold higher than normal in the spleen and 43 fold above normal in the gills of fish infected with *Y. ruckeri* at 96 h post-infection. The expression of asCATH1 was upregulated to a lesser extent, peaking at 30 fold higher in the spleen than controls at 72 and 96 h post-infection and at a maximum of 22 fold in the gills, which is comparable with expression studies of the Atlantic cod cathelicidin. At its peak expression the codCATH was upregulated 32 fold compared to 0 h uninfected fish in the spleen and 10 fold upregulated in the head kidney [35]. Although, studies in mice have seen the mouse cathelicidin, CRAMP, expressed as much as 1200 fold higher than controls in the lungs of mice 28 d post-infection during progressive tuberculosis [38], this study suggested the magnitude of the response may have been due to the high bacterial load and route of infection (injection of 2.5×10^5^ *Mycobacterium tuberculosis* cells). Mammalian studies have reported cathelicidin expression changes in the order of 2–3 fold increases as a normal response during bacterial infection compared to uninfected controls, including the porcine cathelicidins PR-39 and protegrin [39] and the human cathelicidin LL-37 [21]. Another group of salmonid AMPs, the rainbow trout beta defensin genes, were upregulated at a maximum fold increase of 16 times that of control fish during infection with *Y. ruckeri* [34].

This study is the first to look at the expression of Atlantic salmon cathelicidins, and indeed any fish cathelicidin, after infection with *Y. ruckeri*. The codCATHs were upregulated in the gills of cod post-infection with *A. salmonicida*, but were not upregulated during infection with *V. anguillarum* [40], indicating that the host cathelicidin response is specific to the bacteria causing the infection. Different strains of *Y. ruckeri* also vary in virulence and produce different disease outcomes [37]. The extent of cathelicidin response seen during yersiniosis may therefore be specific to the Tasmanian O1b serotype of *Y. ruckeri*. Further studies are needed to compare the relative levels of asCATH1 and 2 expression seen in this study with expression during infection with other strains of *Y. ruckeri* as well as other diseases of Atlantic salmon.

### Antibacterial properties of Atlantic salmon cathelicidins and LL-37

**Figure 3:**
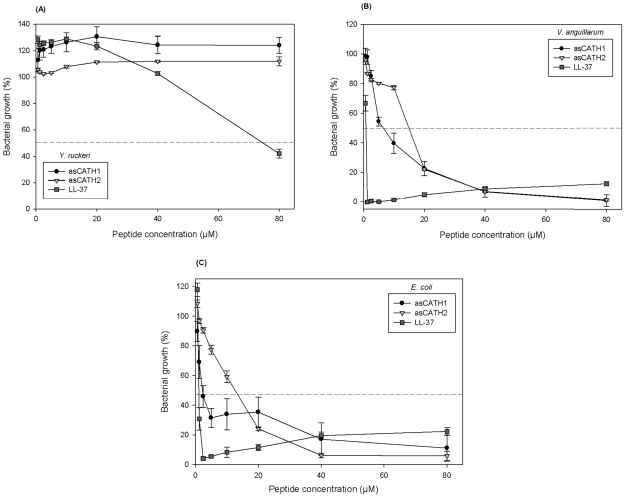
Antibacterial activity of asCATH1 and 2 against three species of bacteria - *Y. ruckeri* (A), *V. anguillarum* (B) and *E. coli* (C). Bacteria were grown to mid-log phase then incubated for 18 h with varying concentrations of the Atlantic salmon cathelicidins. LL-37 was included as a control for bacterial killing and *E. coli* ATCC 25922 was included as a control bacterium. Absorbance was read at 600 nm and percentage growth was calculated by comparison to the no inhibition control lane in the assay. The MIC was defined as 50% inhibition, which is indicated by a dotted line on each plot. Data shown are the mean ± SE of triplicate wells.

Both asCATH1 and 2 were antimicrobial to *V. anguillarum* at relatively low MICs, however neither peptide showed any activity to *Y. ruckeri* up to 80 µM (Fig. 3). LL-37 had an MIC of 40–80 µM to *Y. ruckeri*, which was much higher than the MICs observed against *V. anguillarum* and *E. coli* (Table 1). Chang et al. [2] reported an MIC of 8 µM for both rainbow trout cathelicidins against *Y. ruckeri* strain MT252. Similarly, both rtCATH1 and 2 were approximately 10 fold more inhibitory towards *V. anguillarum* O1 strain (MT1742) than asCATH1 or 2 were to the O1 strain used in the present study. Interestingly, Chang et al. [2] reported an MIC of 1 µM against the control strain of *E. coli* (ATCC 25922) which was very similar to the MIC of 1.25–2.5 µM we found for asCATH1 using the identical *E. coli* strain. The mature cathelicidin peptides are highly variable, which may explain why cathelicidins of relatively closely related species within the salmonidae family, show such variation. The inability to clear bacteria and thus the continual presence of a stimulus for expression may explain the prolonged upregulation of the cathelicidins observed in this study. However, many factors are involved in immune responses and the pathways by which cathelicidins are induced and controlled in fish are unknown. High antibacterial activity is often correlated with haemolytic activity of cathelicidins [41]. The asCATH1 peptide showed more potent antimicrobial activity than asCATH2, which corresponded to the toxicity of the two peptides. When exposed to asCATH1 an MIC of 1.25–2.5 µM against the control strain of *E. coli* was observed, while asCATH2 had a much higher MIC of 10–20 µM. This variance in antimicrobial activity between peptides is typical of multi-cathelicidin species, such as the chicken, sheep and rainbow trout [2], [7], [42]. The human peptide LL-37 displayed similar antimicrobial activity to *E. coli* as asCATH1 with an MIC of 0.63–1.25 µM, which is close to previously reported MICs for LL-37 to the ATCC 25922 strain of *E. coli* (0.1–0.8 µM) [43].

**Table 1:** Antibacterial activity of asCATH1 and 2.

Bacteria	asCATH1	MIC (µM) asCATH2	LL-37
***Y. ruckeri***	>80	>80	40–80
***V. anguillarum***	5–10	10–20	0.63–1.25
***E. coli*** ** (ATCC 25922)**	1.25–2.5	10–20	0.63–1.25

Bacteria were incubated with varying concentrations of cathelicidin peptides for 18 h before absorbance was read at 600 nm to determine bacterial growth. MIC values are shown as the range of concentrations which produced a 50% reduction in growth compared to a control without cathelicidin.

### Haemolytic activity of Atlantic salmon cathelicidins and LL-37

**Figure 4:**
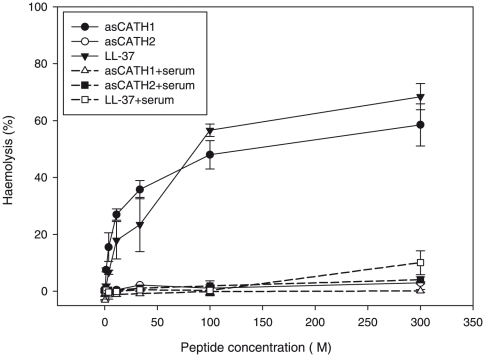
Haemolytic activity of the Atlantic salmon cathelicidins to Atlantic salmon erythrocytes. Erythrocytes were incubated with varying concentrations of the cathelicidins for 2 h and the absorbance of the well supernatants was read at 405 nm to detect released haemoglobin. Percentage haemolysis was calculated from 100% lysis controls, which were incubated with 0.2% Triton-X 100. Data shown are the means ± SD of duplicate wells.

Typical signs of yersiniosis in Tasmanian farmed Atlantic salmon include haemorrhaging around the pectoral and pelvic fins, blood spots in the eyes and hypertrophy of the spleen [33]. Other cathelicidins are haemolytic to host erythrocytes and cytotoxic to other cell types [41], [44], thus in an attempt to reveal possible reasons behind the extensive haemorrhaging that occurs during yersiniosis we investigated the haemolytic activity of asCATH1 and 2. asCATH2 showed no haemolytic activity to Atlantic salmon erythrocytes, while asCATH1 and LL-37 were haemolytic to Atlantic salmon erythrocytes (Fig. 4). asCATH1 caused haemolysis of Atlantic salmon erythrocytes at concentrations as low as 1.24 µM, where haemolysis was 7.54%, and up to 58% haemolysis at higher concentrations. In contrast the haemolytic activity of asCATH1 and LL-37 was absent in the presence of Atlantic salmon serum at all concentrations apart from the highest. Even at the highest concentration of 300 µM the haemolytic activity of asCATH1 and LL-37 was dramatically reduced from 58% to 4% and 60% to 10%, respectively. rtCATH1, which belongs to the salmonid CATH1 clade, caused only 2% haemolysis at 256 µM and less than 10% haemolysis at concentrations as high as 2560 µM [6]. Differences in the haemolytic activity of cathelicidin family members have been observed in sheep where SMAP-29 was more haemolytic than SMAP-28 [41], [44]. The difference in haemolytic activity between asCATH1 and 2 may be due to the structural differences in the peptides – the presence or absence of a disulphide bond in the salmonid CATH1 and CATH2 mature peptides, respectively.

Cathelicidins are amphipathic in nature and cause cell lysis by destabilising membranes and subsequent cell rupture. A stronger electrostatic interaction with prokaryotic membranes than eukaryotic makes cathelicidins selective in killing bacteria rather than damaging self-cells [41], [45]. Basañez et al. [45] demonstrated this with the hagfish cathelicidins, which associated with model bacterial membranes to a much greater extent than erythrocyte membranes. However, mechanisms other than the electrostatic interaction between peptide and membrane are involved in conferring selectivity of non-self, for instance membrane composition and additional properties of the peptide such as hydrophobicity [46]. In this study the peptide-membrane interaction between asCATH1 and Atlantic salmon erythrocytes creates membrane instability that was observed as haemolysis in the haemolytic assay. Therefore, asCATH1 possibly contributes to the signs of yersiniosis in Atlantic salmon through its potential cytolytic actions. Interestingly, we found that serum from Atlantic salmon blood inhibited the haemolytic activity of asCATH1 suggesting that haemolytic activity is unlikely to occur *in vivo* and that potential cytolytic activity would be limited to tissues and serum-free epithelial surfaces. Similarly, *in vitro* tests of other cathelicidins have shown that serum inhibits haemolytic/cytotoxic and antibacterial activity [47], [48]. While we demonstrated that haemolytic activity was reduced in the presence of serum further research is required to assess the potential inhibitory effect of serum on the antibacterial activity of asCATH1 and 2 peptides.

### Host immunomodulatory activities of Atlantic salmon cathelicidins

**Figure 5:**
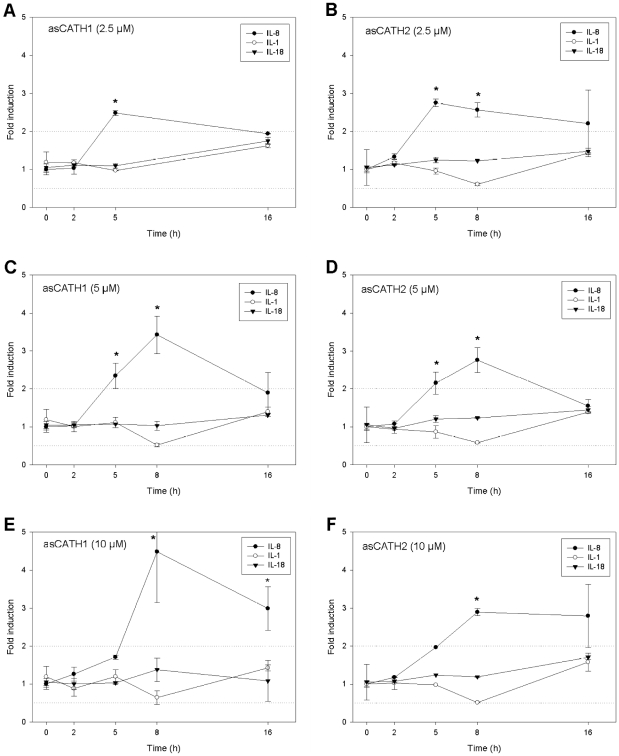
Kinetic profile of IL-8, IL-1 and IL-18 gene expression in Atlantic salmon peripheral blood leukocytes stimulated with asCATH1 (A,C,E) and asCATH2 (B,D,E). Gene expression was assessed by real-time PCR at 0, 2, 5, 8 and 16 h post incubation with asCATH1 or asCATH2 at 2.5 µM (A,B), 5 µM (C,D) and 10 µM (E,F). Expression was normalised to the mean expression of four stable reference genes. Data shown are the means ± SE of quadruplicate PBL samples assayed in duplicate by quantitative real-time PCR and presented as fold induction compared to 0 h. * indicates a significant fold induction (P<0.05) compared to 0 h while the dotted line represents the minimum fold change (2 fold) deemed biologically significant.

Despite showing no inhibition of *Y. ruckeri* in the broth microdilution assay, asCATH1 and 2 were significantly upregulated during the immune response in yersiniosis – up to 98 fold higher than expression observed in uninfected fish (Fig. 2). The expression of asCATH1 was significantly less than asCATH2 in response to *Y. ruckeri* and showed a more restricted constitutive expression profile in the organs tested in this study. This differential expression may help protect the host from the potential cytolytic effects of asCATH1. Furthermore, asCATH1 showed haemolysis at concentrations as low as 1.24 µM, a concentration that is reasonable to expect *in vivo*. The human cathelicidin LL-37 is maintained at a physiological concentration of approximately 0.44 µM (2 µg/mL) [21], [49], [50]. Thus if asCATH1 is present at a similar physiological concentration as LL-37, a 3 fold increase in peptide expression is all that is needed to reach a haemolytic concentration. However, asCATH2 showed no haemolysis and less antibacterial activity than asCATH1. Therefore the question arises as to what function does the increased expression of asCATH2 fulfil? Recent mammalian studies provide convincing evidence that some AMPs do not simply kill microbes but indirectly confer protection by modulating the immune system [24], [51], [52], [53]. Likewise, we demonstrate that both asCATH1 and 2 stimulated the increased expression of the chemokine interleukin-8 (IL-8) in peripheral blood leukocytes (PBLs). To investigate the influence of asCATH1 and 2 on cytokine production we stimulated PBLs for 16 h with varying concentrations of the peptides and measured transcriptional changes in interleukin-1 β (IL-1 β), interleukin-18 (IL-18), and IL-8 expression, of which the latter has been reported to be regulated by cathelicidins in humans [21], [54]. IL-8 expression was significantly upregulated at either 5 or 8 h post-stimulation at all concentrations (2.5 µM, 5 µM, 10 µM) but was greatest (4–5 fold) in PBLs stimulated with 10 µM asCATH1 (Fig. 5). The increased IL-8 mRNA expression was transient as it was reduced to levels deemed biologically insignificant (2 fold or less) by 16 h in all but the PBLs stimulated with asCATH1 at 10 µM. Furthermore, neither asCATH was found to differentially regulate the expression of the pro-inflammatory cytokines IL-1 or IL-18. The transient and specific stimulation of the chemokine IL-8 supports the argument that asCATHs likely act to signal the recruitment of immune cells via IL-8 to the site of infection to help resolve the infection. These immunomodulatory properties of cathelicidins are of particular interest in current mammalian research for use as templates in the design of future antibiotics or adjuvants for vaccination [49], [55], [56]. Thus the development of artificial analogues of these natural peptides will be invaluable not only for mammalian use, but also in aquaculture as potential adjuvants for vaccines.

In summary, we demonstrated that asCATH2 in contrast to asCATH1 was constitutively expressed in healthy uninfected Atlantic salmon and showed that the expression of both was induced during infection with *Y. ruckeri*. Interestingly, the potent antimicrobial activity of both cathelicidins against *E. coli* and the fish pathogen *V. anguillarum* was non-existent when tested against *Y. ruckeri*. Furthermore, we discovered that the constitutively expressed asCATH2 displayed no host haemolytic activity and that the haemolytic activity of asCATH1 was substantially reduced in the presence of host serum. Further supporting the potential multifaceted biological roles of these cathelicidins, especially the constitutively expressed asCATH2, is our finding that asCATH1 and asCATH2 up-regulated the expression of the chemokine interleukin-8 and thus possibly promoted cell migration to the site of infection. This property is in addition to the direct antimicrobial abilities of asCATH1 and asCATH2 and suggests that cathelicidins perform an elaborate role in combating infection and one that possibly circumvents pathogen resistance by effectively utilising the increasingly important relationship between the innate and adaptive immune systems.

## Materials and Methods

### Ethics statement

All animal work was performed in strict accordance with the Australian Code of Practice for the Care and Use of Animals for Scientific Purposes and was approved by the University of Tasmania Animal Ethics Committee (AEC permit number: A0010335).

### Fish husbandry and sampling

Atlantic salmon (*Salmo salar*) parr weighing 4–5 g were obtained from a local hatchery (SALTAS, Wayatinah, Tasmania, Australia) and maintained in a semi-recirculating freshwater system at 15°C. Fish were euthanized and tissue dissected from the gill, spleen, anterior kidney, posterior kidney, liver, intestine, pyloric caeca, heart, skeletal muscle, brain, and stored in an RNA preservation reagent (25 mM sodium citrate, 10 mM EDTA, 10 M ammonium sulphate, pH 5.2) at −20°C until use.

### Constitutive expression of Atlantic salmon cathelicidin genes

Total RNA was extracted from tissue stored in an RNA preservation reagent and purified using TRI Reagent (Molecular Research Center, OH, USA) including DNAse treatment (Turbo DNase, Ambion, TX, USA). First strand cDNA was synthesised from total RNA (1 µg) using BioScript reverse transcriptase (Bioline, NSW, Australia) with Oligo (dT)18 priming according to manufacturer's instructions. asCATH1 and asCATH2 were amplified by PCR to detect expression of the cathelicidins in cDNA from a range of organs from uninfected Atlantic salmon. Primers were designed using Primer Premier 5.0 design software (Biosoft International, CA, USA) from the asCATH1 and 2 sequences published in GenBank with accession numbers AY728057 and AY360357, respectively. The housekeeping gene β-actin was also amplified to assess the quality and allow semi-quantitative analysis of the cDNA samples. All PCR reactions were performed in 20 µl reaction volumes using Immomix (Bioline), 0.5 µl forward primer (10 µM), 0.5 µl reverse primer (10 µM) and 7 µl water. The PCR reactions were run for 35 cycles using the quantitative real-time PCR (qPCR) cycling protocol described below and visualised on a 1% lithium borate agarose gel containing Gel Red dye (Biotium, CA, USA) under UV light.

### Bacterial challenge and cathelicidin expression

Fish were challenged with bacteria by 1 h bath exposure to 1×10^7^ cfu/mL of *Yersinia ruckeri* strain TCFB 2282. At various time points post-infection (0, 8, 24, 48, 72 and 96 h) 6 fish were euthanized and gills and spleen were sampled and stored in RNA preservation reagent at −20°C until use. Quantitative real-time PCR (qPCR) was performed on cDNA reverse-transcribed from total RNA as described above from gill and spleen samples from fish at different time points post-challenge and used to analyse changes in asCATH1 and 2 expression over time. The relative expressions of asCATH1 and 2 were measured by qPCR using SYBR Green chemistry using an iQ5 Real-time PCR Detection System (Bio-Rad, NSW, Australia). Table 2 lists the primers for asCATH1 and 2 detection and of the four reference genes - β-actin, RNA polymerase 2 (RPL2), polyubiquitin (PolyUb) and elongation factor 1α (EF1a). qPCR reactions consisted of 20 µl volumes using a 2×SensiMixPlus SYBR & Fluorescein PCR master mix (Bioline), forward and reverse primers (200 nM of each) and 2 µl of cDNA. Each gene was assayed in duplicate and a five step, four-fold dilution series of a pool of cDNA from all samples was included on the same plate to calculate amplification efficiencies. The amplification program was as follows: 95°C for 10 min to activate the DNA polymerase followed by 40 cycles of 95°C for 10 s, 55°C for 15 s and 72°C for 15 s. At the end of the cycling protocol melt curve analysis was run to ensure amplification specificity. mRNA expression levels were normalised using the mean expressions of the four reference genes, which maintained stable expression, as determined by the geNorm software [57]. The qPCR data were analysed with qBase software as described [58]. A one-way analysis of variance (ANOVA) with Dunnett's post-test was used to compare the expression of asCATH1 or 2 in the gill and spleen at each time point post-infection to a control group (0 h samples). All statistics were performed in SigmaPlot 11.0 (Systat Software, CA, USA) with a P value <0.05 indicating significant increases in gene expression. Data was graphed using SigmaPlot 11.0 and rescaled so that the MNRQ of the 0 h group was equal to 1 and therefore the other time points were presented as a fold increase compared to 0 h. As the data were rescaled, the standard errors were propogated as described by [59]).

**Table 2:** Oligonucleotide primers used in real-time pcr experiments.

Target mRNA	Accession #	Forward primer (5′→3′)	Reverse primer (5′→3′)
asCATH1	AY728057	CTTAATTGGTCGCCTG	ACGTTGATCTGTGATAAAT
asCATH2	AY360357	TGGCAACACCCTCAA	GAATCTTTACTACCCATCT
β-actin	AF012125	TTGCGGTATCCACGAGAC	TAGAGGGAGCCAGAGAGG
RPL2	BT056485	TAACGCCTGCCTCTTCC	ATGAGGGACCTTGTAGCC
PolyUb	BT049644	TCTTCATCTGGTCCTGC	AATGGGTGGGATTGGAGG
EF1a	AF321836	TGATTGTGCTGTGCTTA	AACGCTTCTGGCTGTAGG
IL-8	BT046706	ACCAGCGAGATAACAA	CCAGGAGCACAATGACAA
IL-1	AY617117	AACTAAGGACTGAATA	GAGGTGTTCTTTATTAAAC
IL-18	BT125392	AACGGAATAAGGAGCT	TGATGTCACAGAGAGAAT

### Peptide synthesis

Two peptides, referred to as asCATH1 and asCATH2, corresponding to the first 36 amino acids of each mature peptide of the previously reported Atlantic salmon cathelicidins [2] were chemically synthesised by Auspep (Tullamarine, Victoria, Australia). Residues 145 to 180 of the *S. salar* cathelicidin (acc. #: AAW55907) was referred to as asCATH1 (RRSQARKCSRGNGGKIGSIRCRGGGTRLGGGSLIGR) and had a molecular weight of 3685. Residues 150 to 185 of the *S. salar* cathelicidin-derived antimicrobial peptide 2 precursor (acc. #: AAT44537) was referred to as asCATH2 (RRGKPSGGSRGSKMGSKDSKGGWRGRPGSGSRPGFG) and had a molecular weight of 3632. To compare the activities of Atlantic salmon cathelicidins with those of a mammalian cathelicidin the extensively researched LL-37 peptide was also synthesized. The LL-37 peptide corresponded to residues 134 to 170 (LLGDFFRKSKEKIGKEFKRIVQRIKDFLRNLVPRTES) of the sole human cathelicidin known as cationic antimicrobial protein 18 (hCAP18, acc. #: NP_004336) and had a molecular weight of 4493. Certificates of analysis provided with each peptide displayed HPLC chromatogram and mass spectral analysis identifying the purity of each peptide– asCATH1 (86%), asCATH2 (97%) and LL-37 (90%).

### Bacteria and culture conditions

Three species of bacteria were used to test the antibacterial activities of the two Atlantic salmon cathelicidins. The Tasmanian O1b serotype of *Y. ruckeri* (UTYR 001A), *Vibrio anguillarum* serotype O1 (UTVA 001) and *Escherichia coli* (ATCC 25922) were maintained on tryptic soy agar plates until use. Individual bacterial colonies were selected from the plates and transferred to 4 mL of Mueller-Hinton broth (MHB). The bacteria were grown for 2–3 h on a shaker at 20°C until they reached mid-log phase. 1 mL of the log-phase suspension was centrifuged at 1,000 xg for 5 min at room temperature and the bacterial pellet was resuspended in 1 mL of one quarter strength (0.25x) MHB. The suspension was diluted 1∶1000 by adding 10 µl to 10 mL of 0.25x MHB to give a final working suspension of approximately 10^5^ cfu/mL. 100 µl of each of a tenfold dilution of the 1∶1000 working suspension (1/10, 1/100 and 1/1000) was spread onto Mueller-Hinton agar plates to count colony forming units (cfu).

### Antimicrobial and haemolysis assays

A broth microdilution assay was used to measure the antibacterial activity of the Atlantic salmon cathelicidins as previously described [60]. Briefly, each peptide was serially diluted in sterile water to give final concentrations of 800, 400, 200, 100, 50, 25, 12.5 and 6.25 µM. To each well of a flat bottom 96 well polystyrene microtitre plate 90 µl of the 1∶1000 bacterial suspension was added. 10 µl of the peptide dilutions were then added to the wells in triplicate so that final peptide concentrations were 80, 40, 20, 10, 5, 2.5, 1.25 and 0.63 µM. Controls were also included in the assay: 10 µl of ampicillin (1 mg/mL) was added to one lane as a control for bacterial killing; a no-inhibition control contained bacterial suspension with 10 µl of water; and a no-growth control contained 100 µl of 0.25x MHB only. Plates were incubated at 20°C for 18 h then absorbances were read at 600 nm. Bacterial growth in wells containing peptide was compared to the no-inhibition control wells (considered 100% growth) to determine the MIC of each peptide. The MIC was defined as the lowest concentration of peptide that reduced bacterial growth by more than 50% compared to control wells [2], [6], [61].

The haemolytic activities of the Atlantic salmon cathelicidins were assessed as described [6], [62]. Briefly, 2 mL of heparinised whole blood was collected from uninfected Atlantic salmon and centifuged at 1,000 xg for 10 min at room temperature. The serum and buffy coat were removed from the tube and discarded. Erythrocytes were then washed in 1 mL of PBS three times, centrifuging at 1,000 xg for 10 min and discarding supernatant between washes. The final pellet was resuspended to 10^8^ cells/mL in PBS or Atlantic salmon serum to assess the effect of serum on the haemolytic activity. Atlantic salmon serum was obtained from whole blood left to clot overnight at 4°C, centrifuged at 3,000 xg for 15 min at 4°C to remove any residual cells, before 0.2 µM filtration. Each peptide was serially diluted in 1x PBS to give concentrations of 3000, 1000, 333.3, 111.1, 37, 12.4, 4.1 and 1.4 µM. 180 µl of the erythrocyte suspension was added to the desired wells of a V-shaped 96 well microtitre plate. 20 µl of each concentration of the cathelicidin peptides was added to wells in duplicate, such that the final peptide concentration was tenfold less than the starting. 20 µl of 2% Triton-X 100 or PBS was added to the control wells for 100% and 0% lysis, respectively. The plates were incubated for 2 h at 18°C then centrifuged for 10 min at 1,000 xg. 100 µl of the supernatant from each well was transferred to a fresh flat-bottom microtitre plate and the optical density was measured at 405 nm. Percentage haemolysis was determined for each concentration of peptide by comparison to 100% and 0% lysis using the following formula: [(A_Cathelicidin_ – A_0% lysis_)/(A_100% lysis_ – A_0% lysis_)]×100, where A is the absorbance at 405 nm.

### Peripheral blood leukocyte (PBL) stimulation

Heparinised whole blood from uninfected Atlantic salmon was diluted 1∶1 with 1x HBSS. A discontinuous Percoll gradient was prepared using Percoll – 1.07 g/mL (49% Percoll) and 1.05 g/mL (34%). Diluted blood was layered onto the Percoll gradient and centifuged at 2,000 xg for 20 min at 4°C. Two bands of cells were removed; one from the serum-34% interface and one from the 34%–49% interface. The collected bands were relayered onto a fresh 34/49% gradient and centifuged at 800 xg for 10 min at 4°C to remove residual erythrocytes. A clear band was removed from the 34%–49% interface and diluted 1:1 with 1xHBSS. The cells were centrifuged at 500 xg for 5 min at 4°C, the supernatant was decanted and cells were resuspended in 1x HBSS. Cells were counted and 100 µl of the cell suspension was also used for a cytospin to confirm the cell population was peripheral bloods leukocytes (PBLs).

PBLs were centrifuged at 350 xg for 5 min and resuspended to a final concentration of 1×10^7^ cells/mL in L-15 culture media containing L-glutamine and 2% Atlantic salmon serum. Peptides were serially diluted in 1xPBS to give final concentrations of 110, 55, 27.5 and 13.75 µM. To each well of a flat bottom 96 well microtitre plate 100 µl of cells in media was added, so that there were approximately 1×10^6^ cells per well. 10 µl of each peptide concentration was added in quadruplicate (giving final well concentrations of 10, 5, 2.5 and 1.25 µM for asCATH1 and 2) for four different sampling time points (2, 5, 8 and 16 h) after peptide addition. No-stimulation controls were included for each time point, with 10 µl of PBS instead of peptide. A 0 h sample was taken directly before plate incubation by adding 110 µl of 2x Tissue and Cell (T&C) lysis solution (Epicentre, WI, USA) to four wells containing cells with PBS. The cells were mixed with the T&C lysis solution before being transferred to a 1.5 mL microcentrifuge tube. 1 mL of Tri-reagent (Molecular Research Center) was added to each tube and samples were vortexed then frozen at −70°C until use. Four samples of cells were taken for each concentration of peptide in the same way after 2, 5, 8 and 16 h incubation at 18°C. Total RNA was extracted, reverse transcribed and cDNA analysed by qPCR as previously described using primers specific for interleukin-1(IL-1), interleukin-8 (IL-8) and interlukin-18 (IL-18) (Table 2).
